# Cloning and functional verification of endogenous U6 promoters for developing an efficient CRISPR/Cas9-mediated genome editing system in kenaf (Hibiscus cannabinus L.)

**DOI:** 10.21203/rs.3.rs-8660152/v1

**Published:** 2026-02-06

**Authors:** Shaolian Jiang, Fangzhou Chen, Haixiong Ma, Siyan Wu, Xin Tang, Xueqing Pan, Qin Li, Aifen Tao, Jiantang Xu, Jianmin Qi, Pingping Fang, Jikang Chen, Liwu Zhang

**Affiliations:** Fujian Agriculture and Forestry University; Fujian Agriculture and Forestry University; Fujian Agriculture and Forestry University; Fujian Agriculture and Forestry University; Fujian Agriculture and Forestry University; Fujian Agriculture and Forestry University; Fujian Agriculture and Forestry University; Fujian Agriculture and Forestry University; Fujian Agriculture and Forestry University; Fujian Agriculture and Forestry University; Fujian Agriculture and Forestry University; Chinese Academy of Agricultural Sciences; Fujian Agriculture and Forestry University

**Keywords:** Kenaf, U6 promoter, CRISPR/Cas9, gene editing

## Abstract

The U6 promoter plays a pivotal role in the CRISPR/Cas9 system by driving the transcription of single guide RNA (sgRNA), which directs Cas9 to achieve precise genome editing. Endogenous U6 promoters typically exhibit superior transcriptional activation efficiency compared to exogenous counterparts, thereby enhancing the efficacy of genome editing. However, the endogenous U6 promoter in kenaf (*Hibiscus cannabinus* L.) remains uncharacterized. In this study, we conducted a homologous search of the kenaf genome using the *Arabidopsis* U6 (AtU6–26) RNA sequence as a reference, identifying two candidate promoters, HcU6–1 and HcU6–14. Promoter fragments were amplified from the genomic DNA of kenaf cultivar ‘Fuhong 952’ and subsequently cloned into a GUS fusion expression vector. Histochemical staining revealed transcriptional activity for both promoters, with HcU6–14 demonstrating significantly stronger activity. To evaluate editing efficiency, we constructed a CRISPR/Cas9 vector containing *HcALS* sgRNA, driven by either the kenaf U6–14P promoter or the cotton U6–9P (GbU6–9P) promoter. Kenaf hairy roots were regenerated via *Agrobacterium* rhizogenes K599-mediated transformation. Sequencing analysis of *ALS* gene fragments from these hairy roots confirmed successful targeted editing when using the kenaf U6–14P promoter, whereas no base mutations were detected with the cotton U6 promoter. These findings highlight the superior editing efficiency of the kenaf U6 promoter and provide a critical foundation for advancing functional genomics research in kenaf.

## 1. Introduction

Kenaf (*Hibiscus cannabinus* L.), an annual herbaceous plant in the Malvaceae family, ranks among the most economically important bast fiber crops globally. Its fibers are renowned for exceptional softness, high tensile strength, and superior hygroscopicity, endowing them with broad application potential in textiles, composites, and sustainable materials[1]. Since its inception in 2013, CRISPR/Cas9 technology has emerged as a transformative genome-editing tool, enabling site-specific modifications—including gene knockout, replacement, and insertion—across plant genomes. Its widespread adoption stems from unparalleled advantages such as high efficiency, cost-effectiveness, and operational simplicity[2].

The endogenous high-efficiency U6 promoter is a key regulatory element for CRISPR / Cas9 system to achieve efficient gene editing. As the core molecule in the CRISPR / Cas9 system that determines the efficiency of targeted recognition and cleavage, the transcription level of sgRNA is usually significantly associated with editing efficiency[3]. Although the CRISPR / Cas9 system has been configured and applied in a variety of plants, the editing efficiency of different species and different genetic transformation systems still shows significant differences[4]. Studies have shown that using the endogenous U6 promoter of the target species to drive sgRNA transcription is an effective strategy to improve the editing efficiency of CRISPR / Cas9 [5, 6]. In recent years, in phloem fiber crops and similar crops, the screening and optimization of U6 promoters also provide direct evidence for targeting : For example, in linseed ( *Linum usitatissimum* ), by identifying multiple endogenous U6 promoters and using the reporter system to screen high-activity promoters, the candidate promoters can be further truncated to maintain high transcriptional activity, and the editing frequency is higher than that of the exogenous U6 promoter drive system in PDS target gene editing[3]. In sorghum (*Sorghum bicolor*), studies have also shown that endogenous U6 promoter can improve CRISPR / Cas9 editing efficiency and show potential reference value for other gramineous crops[6]. In addition, U6 promoter cloning and transcriptional activity analysis have also been reported in bast fiber crops such as jute (*Corchorus capsularis*), which provides promoter resources and methodological basis for establishing species-specific sgRNA expression modules and optimizing editing vector design for such crops[7].

The U6 promoter is a canonical RNA polymerase III (Pol III) promoter, recognized by Pol III to initiate U6 small nuclear RNA (snRNA) transcription[8]. Transcriptionally active U6 promoters typically harbor two conserved cis-elements: a TATA-like box positioned at approximately − 30 bp and an upstream sequence element (USE) around − 60 bp [9]. Additionally, the transcription start site is invariably defined by a guanine (G) residue, a feature that not only guarantees accurate sgRNA transcription but also significantly reduces off-target effects by enhancing target-binding specificity[10]. Given the strict species specificity of U6 promoters, identifying endogenous high-activity U6 promoters is imperative for achieving efficient targeted editing [11]. To date, endogenous U6 promoter-driven CRISPR/Cas9 systems have been validated in multiple crops, including Nicotiana tabacum [12], alfalfa[13], Walnut[14], and Fraxinus mandshurica[15]. However, no studies have reported the characterization or application of endogenous U6 promoters in kenaf, nor has successful CRISPR/Cas9-mediated editing been documented for this species. Consequently, the identification of kenaf-specific U6 promoters is urgent for advancing functional genomics and enabling precise genetic improvement.

Acetolactate synthase (ALS; EC 4.1.3.18), also known as acetohydroxyacid synthase (AHAS; EC 2.2.1.6), is a pivotal enzyme in the biosynthesis of branched-chain amino acids (valine, leucine, and isoleucine) in plants and microorganisms[16]. nhibition or loss of *ALS* activity disrupts amino acid synthesis, leading to growth inhibition, developmental defects, or even plant death[17]. Exploiting this mechanism, *ALS* has become a classical target for highly effective herbicides, including imidazolinones (IMI) and sulfonylureas (SU)[18].

In this study, we identified two candidate U6 promoters, HcU6–1 and HcU6–14, from the kenaf cultivar ‘Fuhong 952’through homologous cloning. Their transcriptional activity was validated using GUS fusion expression assays, revealing HcU6–14 as the most active promoter. Subsequently, we constructed CRISPR/Cas9 vectors driven by either HcU6–14P or the cotton U6 promoter GbU6–9P (as a control). Using an Agrobacterium rhizogenes-mediated hairy root transformation system, we systematically assessed editing efficiency at *ALS* target sites. This work would provide foundational technical support for developing herbicide-resistant germplasm.

## 2. Materials and Methods

### 2.1. Plant materials

Seeds of kenaf ‘Fuhong 952’ and *Nicotiana benthamiana* were maintained in the Laboratory of Genetic Breeding and Comprehensive Utilization of Bast Fiber Crop, Fujian Agriculture and Forestry University. Competent cells of *Agrobacterium tumefaciens* GV3101, *Agrobacterium rhizogenes* K599, *Escherichia coli* DH5α, and *E. coli* TOP10 were purchased from Shanghai Weidi Biotechnology Co., Ltd. Plasmids pRNTR, pGWB533, p533–35S::GUS, and pRGEB32 were preserved in the same laboratory. LR Clonase enzyme mix was obtained from Invitrogen (Thermo Fisher Scientific, UK). High-fidelity DNA polymerase was purchased from TaKaRa (Japan). GoTaq^®^ qPCR Master Mix was purchased from Promega (Beijing, China). The GUS staining kit was obtained from Huayueyang Biotechnology (Beijing, China). The seamless cloning kit and gel extraction kit were purchased from Vazyme Biotech Co., Ltd. (Nanjing, China). The RNA extraction kit, RNA reverse transcription kit, plant genomic DNA extraction kit, and plasmid miniprep kit were purchased from Tiangen Biotech (Beijing, China). Quantitative real-time PCR was performed using a CFX96 Real-Time PCR Detection System (Bio-Rad, USA), and sequencing as well as primer synthesis were conducted by Fuzhou Shangya Biotechnology Co., Ltd.

### 2.2. Cloning of HcU6 promoter and construction of HcU6 : : GUS fusion expression vector

Candidate HcU6 snRNA genes were identified in a kenaf genome database assembled in our laboratory using the conserved *Arabidopsis thaliana* U6 snRNA sequence as a query. The corresponding upstream promoter regions were retrieved for each candidate HcU6 RNA locus. After 5′ truncation of the *U6* promoter sequences, PCR primers were designed for amplification (Table 1). Target fragments were amplified using a high-fidelity DNA polymerase (TaKaRa; Lot No. AO11054A; Japan). PCR products were verified by 1% agarose gel electrophoresis and subsequently purified using a gel extraction kit (Tiangen; Lot No. A0750B; China).

Purified fragments were recombined into the pRNTR vector using a seamless cloning kit according to the reaction system shown in Table 2, and incubated at 37°C for 30 min for homologous recombination. The recombination products were introduced into competent *Escherichia coli* DH5α cells by heat shock. Cells were recovered at 37°C with shaking at 200 rpm for 30 min, plated on selective medium, and incubated overnight at 37°C. Single colonies were screened by colony PCR; positive clones were sequenced using primer HcU6–14P (Table 1). Sequence alignment confirmed correct assembly, indicating successful construction of the intermediate vector HcU6::pRNTR.

Plasmids were isolated from sequence-verified positive clones. The entry vector contained the target promoter fragment flanked by attL1 and attL2 sites, whereas the destination vector pGWB533 carried the GUS reporter gene and attR1 and attR2 sites. An LR recombination reaction was performed using LR Clonase (Invitrogen) at 25°C following the pRNTR-U6 reaction system (Table 3), with incubation at 16°C overnight. The LR reaction products were transformed into competent *E. coli* DH5α cells by heat shock. Single colonies were subjected to colony PCR using primers HcU6–1P and HcU6–14P (Table 1). Positive clones were sequenced by Shangya Biotechnology Co., Ltd. (Fuzhou, China), and correct sequence alignment confirmed successful construction of the HcU6::GUS fusion expression vector.

### 2.3. Transcriptional activity identification of HcU6 promoter

The successfully constructed HcU6::GUS fusion expression vectors, together with the positive control p533–35S::GUS and the negative control plant expression vector, were introduced into competent *Agrobacterium tumefaciens* GV3101 cells by heat shock. Transformants were cultured in LB liquid medium supplemented with kanamycin (50 μg mL^−1^), rifampicin (25 μg mL^−1^), and gentamicin (25 μg mL^−1^) with shaking until the bacterial suspension reached an OD_600_ of 0.8–1.0. Cells were harvested by centrifugation at 5000 r min^−1^ for 10 min and resuspended in infiltration buffer (MES, 10 mM; MgCl_2_·6H_2_O, 10 mM; MgCl_2_·7H_2_O, 10 mM; acetosyringone, 100 μM). The suspension was incubated in the dark at 28°C for 2 h and then infiltrated into leaves of 4-week-old *Nicotiana benthamiana*. Each treatment included three biological replicates. After 24 h of co-cultivation, leaf discs were collected using a cork borer. Leaf discs were immersed in GUS staining solution and incubated at 37°C with shaking at 180 r min^−1^ for 12 h, after which the staining solution was removed. The stained discs were destained in 75%–100% ethanol, with the ethanol replaced every 2 h until chlorophyll was completely removed. Destained samples were examined and photographed under a stereomicroscope, and *HcU6* promoters capable of driving GUS expression in tobacco leaves were identified based on staining intensity.

In parallel, the fusion expression vector p533-HcU6::GUS (experimental group) and the p533–35S::GUS plasmid (positive control) were transformed into competent *Agrobacterium rhizogenes* K599 cells. *A. rhizogenes* K599 harboring the empty vector served as the negative control. Kenaf cotyledonary nodes were inoculated with K599 to induce transgenic hairy roots at the wound sites[19]. After 1–2 weeks of growth, regenerated kenaf hairy roots were subjected to GUS histochemical staining. The staining procedure was similar to that used for tobacco; however, because regenerated kenaf hairy roots are white, destaining was unnecessary. Stained hairy roots were directly observed and imaged under a stereomicroscope to evaluate promoter transcriptional activity.).

### 2.4. Construction of CRISPR / Cas9 vector

The pRGEB32-GhU6.9-NPT2 vector was double-digested with HindIII and BsaI, and the linearized backbone was purified by gel extraction. Using genomic DNA from kenaf ‘Fuhong 952’ as the template, the HcU6–14 promoter fragment was amplified with primer HcU6–14P-a containing HindIII/BsaI recognition sites. The purified promoter fragment was inserted into the linearized vector by homologous recombination and subsequently transformed into competent *E. coli* DH5α cells. Positive clones were verified by PCR (using primer HcU6–14P-a) and Sanger sequencing, confirming successful construction of the base vector HcU6–14P::sgRNA-Cas9.

**Figure 1:**
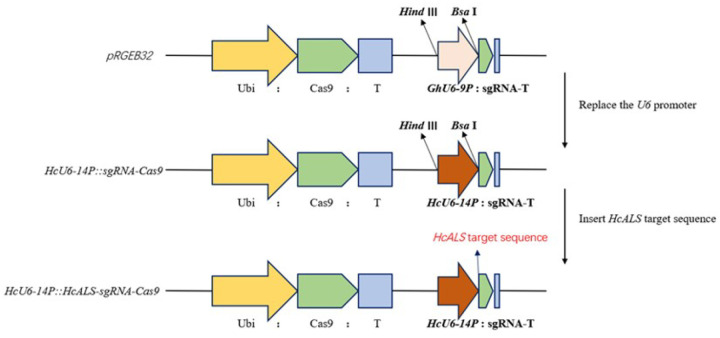
Construction of CRISPR/Cas9 vector in kenaf. The figure illustrates an expression vector driven by the cotton U6 promoter. The middle section shows the construction where the cotton U6 promoter is replaced by the kenaf U6 promoter, and the bottom section represents the *ALS*target gene expressed under the control of the kenaf U6 promoter.

A target site (5′-GCAGTGGTCGTAGACATCGA-3′) was designed within an exon of the kenaf *HcALS* gene and cloned accordingly. This target sequence was incorporated into the sgRNA expression cassette of both the HcU6–14P::sgRNA-Cas9 vector and the GhU6–9P::HcALS-sgRNA-Cas9 vector (Fig. 1).

### 2.5. Hairy root preparation and genetic transformation of kenaf

The kenaf cultivar ‘Fuhong 952’ was used in this study. Seeds were surface-sterilized with 50% 84 disinfectant solution hypochlorite (NaOCl) and 75% ethanol, and then evenly sown on MS medium (4.43 g L^−1^ MS salts, 8 g L^−1^ agar, and 30 g L^−1^ sucrose). Seedlings cultured for 10–14 d were used as explant donors, and cotyledonary nodes were excised for infection.

The construct HcU6–14P::HcALS-sgRNA-Cas9 was used as the experimental group, while GhU6–9P::HcALS-sgRNA-Cas9 served as the positive control. *Agrobacterium rhizogenes* K599 carrying the corresponding plasmids was used for transformation, and K599 without the recombinant vector was used as the negative control. Positive *A. rhizogenes* cultures were propagated in LB liquid medium containing kanamycin (50 μg mL^−1^), rifampicin (25 μg mL^−1^), and gentamicin (25 μg mL^−1^) at 28°C and 200 rpm for 24 h. Cells were collected by centrifugation, resuspended in infection medium (4.43 g L^−1^ MS, 30 g L^−1^ sucrose, 0.2–0.4mg L^−1^ TDZ, 0.1–0.2 mg L^−1^ 2,4-D, 20 mg L^−1^ acetosyringone, 0.2 mg L^−1^ GA3, pH 5.6), and adjusted to an OD_600_ of 0.5. Cotyledonary-node explants were inoculated for 20 min in a laminar-flow hood, blotted dry with sterile filter paper, and transferred to co-cultivation liquid medium (4.43 g L^−1^ MS, 30 g L^−1^ sucrose, 0.1–0.2 mg L^−1^ TDZ, 0.1–0.2 mg L^−1^ 2,4-D, 20 mg L^−1^ acetosyringone, 0.2 mg L^−1^ GA3, pH 5.6) for 48 h in the dark. After co-cultivation, explants were moved to rooting medium (4.43 g L^−1^ MS, 30 g L^−1^ sucrose, 8% agar, 0.1–0.3 mg L^−1^ IAA, 2.5 mL L^−1^ timentin, pH 5.8)[20] to induce hairy root formation. Explants were cultured for 1–2 weeks to obtain regenerated kenaf hairy roots (M).

### 2.6. Sequencing-based detection of mutations at the HcALS target site

Genomic DNA from regenerated transgenic kenaf hairy roots was used as the template for PCR amplification with the vector-specific primer RTCas9 (Table 1). PCR products were examined by 1% agarose gel electrophoresis, and the positive rate was calculated. Genomic DNA from PCR-positive T0 lines was subsequently amplified using the target-site primer pair *ALS* test (Table 1). Purified PCR products were submitted to Shangya Biotechnology Co., Ltd. for Sanger sequencing. Mutation rates and types were analyzed, with positive and mutation rates determined as follows: Positive rate (%) = transgenic hairy roots / regenerated hairy roots × 100; Mutation rate (%) = mutant hairy roots / transgenic hairy roots × 100.

## 3. Results

### 3.1. Cloning and sequence analysis of *HcU6* promoter

**Figure 2:**
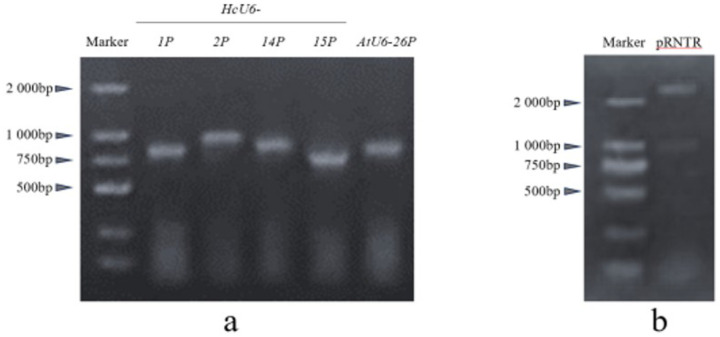
Cloning of U6 Promoters and Digestion of the pRNTR Vector Cloning of *U6* promoters. a The *HcU6–1P* (863 bp), *HcU6–2P*(996 bp), *HcU6–14P* (953 bp), *HcU6–15P* (749 bp), and *Arabidopsis AtU6–26* promoter (911 bp) were successfully cloned.Digestion of the intermediate vector plasmid pRNTR b The HcU6/AtU6 and vector plasmid pRNTR were digested with the restriction enzymes HindIII and BamHI, resulting in two bands of 2241 bp and 1000 bp for the pRNTR plasmid.

A candidate U6 promoter homologous to *Arabidopsis thaliana AtU6–26* was identified by searching the kenaf genome dataset. PCR primers were designed based on 5′-truncated fragments of the aligned candidate U6 promoter sequences. Using a high-fidelity DNA polymerase, four 5′-truncated kenaf U6 promoters were cloned and designated *HcU6–1P, HcU6–2P, HcU6–14P*, and *HcU6–15P*, with lengths of 863, 996, 953, and 749 bp, respectively. In addition, a 911-bp fragment of the *A. thaliana AtU6–26* promoter was successfully cloned (Fig. 2a).

Sequence alignment of the kenaf U6 promoters (*HcU6–1P, HcU6–2P, HcU6–14P*, and *HcU6–15P*) and the A. thaliana U6 promoter was performed using DNAMAN (v6.0). The analysis indicated that the TATA box, the upstream sequence element (USE; 5′-TCCCACATCG-3′), and the spacing between these two elements are key determinants of U6 promoter transcriptional activity. All promoters examined (*AtU6-P, HcU6–1P, HcU6–2P, HcU6–14P*, and HcU6–15P) contained the conserved Pol III promoter elements, namely the TATA box and USE, and the distance between these motifs was relatively constant (Fig. 3). Based on these results, *HcU6–1P* and *HcU6–14P* were selected for subsequent assays of transcriptional activity.

### 3.2. HcU6 : : GUS fusion expression vector and *HcU6* promoter transcriptional activity

Both *HcU6/AtU6* promoter fragments and the pRNTR vector were digested with the restriction endonucleases HindIII and BamHI. After digestion, pRNTR yielded two bands of 2241 bp and 1000 bp (Fig. 2b). The linearized vector backbone was further gel-purified and used for subsequent ligation. Sequencing results were consistent with the expected constructs, confirming successful generation of the HcU6::GUS and AtU6::GUS vectors. The validated HcU6::GUS and AtU6::GUS constructs, together with the positive control p35S::GUS and a negative control, were transiently expressed in tobacco leaves via Agrobacterium tumefaciens–mediated infiltration and were also introduced into kenaf hairy roots through *Agrobacterium rhizogenes* K599–mediated transformation, followed by GUS histochemical staining.

**Figure 4:**
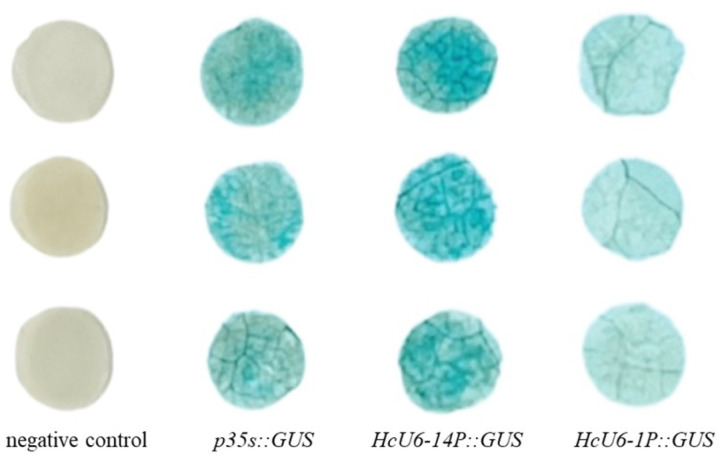
Expression of GUS driven by different *U6* promoters in tobacco leaves. p35S::GUS serves as a positive control. GUS gene expression driven by both *HcU6–14*and *HcU6–1* promoters was observed in tobacco leaves, with blue coloration in both. Leaves with *HcU6–14* showed a deeper blue color than those with *HcU6–1*, indicating stronger activity of the *HcU6–14*promoter.

**Figure 5:**
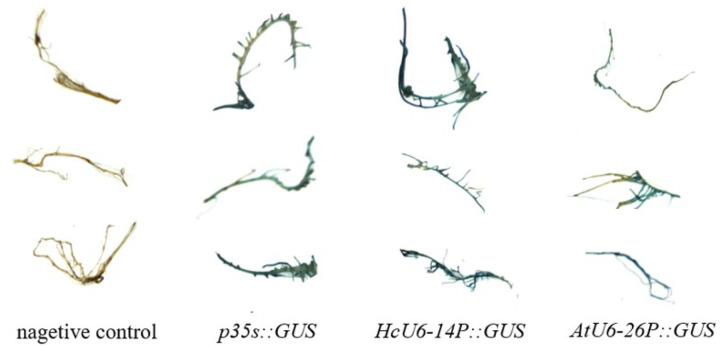
Expression of GUS driven by different *U6* promoters in kenaf hairy roots. The GUS staining results show that both promoters *HcU6–14* and *HcU6–1* exhibit significant activity in the hemp hairy roots, with the roots turning deep blue compared to the negative control. The hairy roots with the *HcU6–14* promoter show a deeper blue color than those with *HcU6–1*, indicating stronger promoter activity for *HcU6–14*.

**Figure 6:**
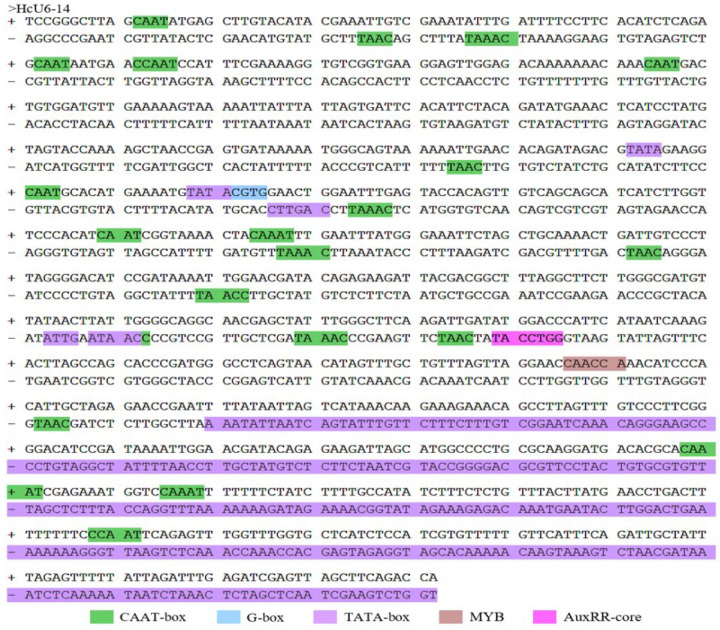
*HcU6–14*promoter sequence cis-element prediction CAAT-box: Homeotropic elements shared by promoter and enhancer regions that bind to transcription factors and regulate gene expression. G-box: Homeopathic elements involved in regulating gene transcription activity. TATA-box: Core promoter elements at the transcription start site. MYB: Homeopathic elements involved in gene expression regulation and multiple biological processes. AuxRR-core: Homeopathic elements involved in plant hormone signal transduction.

In tobacco leaves, both *HcU6–14* and *HcU6–1* were able to drive GUS expression, as evidenced by blue staining of infiltrated leaf tissues. Moreover, staining was visibly stronger for *HcU6–14* than for *HcU6–1* (Fig. 4), indicating higher promoter activity of *HcU6–14*. Consistently, GUS staining in kenaf hairy roots showed the same trend: compared with the negative control, hairy roots transformed with *HcU6–14* or *HcU6–1* displayed intense blue staining, with HcU6–14 again producing a darker signal than *HcU6–1* (Fig. 5). Based on these results, cis-regulatory element prediction was further performed for the *HcU6–14* promoter (Fig. 6). The analysis revealed multiple CAAT-box and TATA-box motifs, as well as one MYB-binding site and one AuxRR-core element within the *HcU6–14* sequence. The CAAT-box is a common regulatory element found in both promoter and enhancer regions and contributes to transcriptional regulation through binding of specific transcription factors. The TATA-box is a core promoter element located near the transcription start site. MYB-related elements are involved in diverse biological processes, including transcriptional regulation of gene expression. The AuxRR-core element is associated with phytohormone signal transduction. Collectively, GUS histochemical assays demonstrated that both *HcU6–14* and *HcU6–1* possess promoter activity, with *HcU6–14* exhibiting higher activity. Therefore, *HcU6–14* was selected for subsequent construction of CRISPR/Cas9 vectors in this study.

The GUS staining results show that both promoters *HcU6–14* and *HcU6–1* exhibit significant activity in the hemp hairy roots, with the roots turning deep blue compared to the negative control. The hairy roots with the *HcU6–14* promoter show a deeper blue color than those with *HcU6–1*, indicating stronger promoter activity for *HcU6–14*.

CAAT-box: Homeotropic elements shared by promoter and enhancer regions that bind to transcription factors and regulate gene expression. G-box: Homeopathic elements involved in regulating gene transcription activity. TATA-box: Core promoter elements at the transcription start site. MYB: Homeopathic elements involved in gene expression regulation and multiple biological processes. AuxRR-core: Homeopathic elements involved in plant hormone signal transduction.

**Figure 7:**
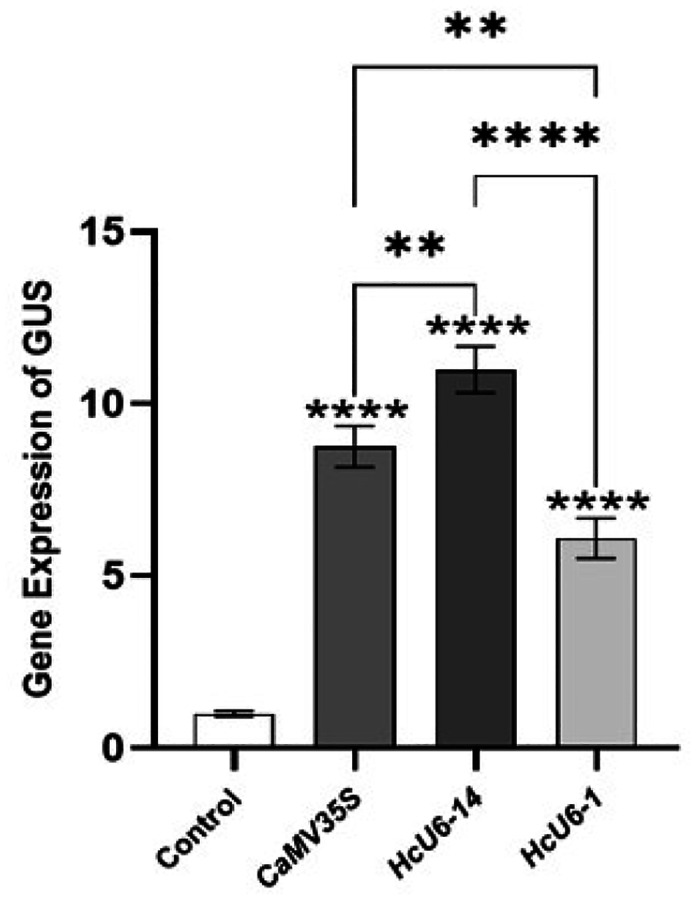
Expression of the GUS gene driven by different U6 promoters in kenaf hairy roots. The negative control showed no GUS expression, and CaMV35S was the positive control. GUS expression driven by *HcU6–14* was significantly higher than *CaMV35S*, while *HcU6–1* showed lower expression. *HcU6–14* had approximately 1.25 times the transcriptional activity of *HcU6–1*. The data represent the transcriptional activities of the three promoters. **, *** indicate significant differences at the 0.01 and 0.001 probability levels, respectively.

Total RNA was extracted from hairy roots emerging at the wound sites of kenaf cotyledonary nodes after infection with *Agrobacterium rhizogenes* K599. Following reverse transcription, transcript levels were quantified by qPCR (Fig. 7). *CaMV 35S* was used as the positive control, whereas no GUS transcript was detected in the negative control. The GUS expression level driven by *HcU6–14P* was significantly higher than that driven by *CaMV 35S*, while the GUS expression level driven by *HcU6–1P* was lower than that of *CaMV 35S*. Moreover, a highly significant difference was observed between the two kenaf promoters. Specifically, GUS expression under *HcU6–14P* was significantly higher than under *HcU6–1P*, indicating that the transcriptional activity of *HcU6–14P* is markedly greater than that of *HcU6–1P*, reaching approximately 1.25-fold of *CaMV 35S*. Collectively, these results demonstrate that the kenaf *U6* promoter *HcU6–14P* exhibits high transcriptional activity and is suitable for subsequent construction of genome-editing vectors.

### 3.3. Construction of a CRISPR/Cas9 vector and mutation detection of the *HcALS* gene

**Figure 8:**
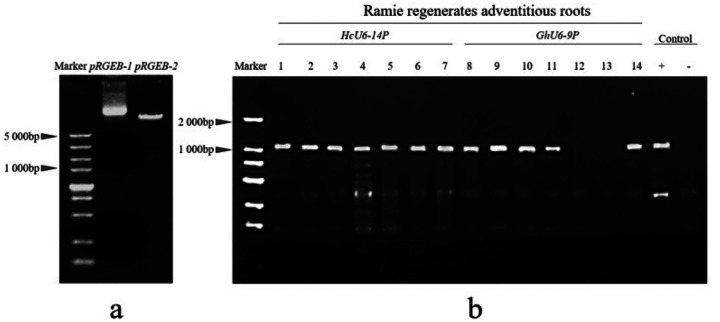
Schematic diagram of the pRGEB-2 vector digested with HindIII and BsaI. (a). After digestion, a 1000bp difference in band pattern was observed for pRGEB32–2 compared to the undigested pRGEB32–1. PCR detection of positive results in regenerated kenaf hairy roots (b). The HcU6–14P::HcALS-sgRNA-Cas9 vector was used for Agrobacterium-mediated transformation of kenaf hypocotyls with strain K599, resulting in 7 transformed regenerated hairy roots. PCR with the universal vector primer RTCas9 confirmed that all 7 strains were positive, yielding a 100% positive rate. The GhU6–9P::HcALS-sgRNA-Cas9 vector was also used for transformation, with Cas9 protein insertion detected in 5 strains (Figure ure 6b), giving a 71.4% positive rate.

The pRGEB32–1 vector was double-digested with HindIII and BsaI. After digestion, the resulting linearized product (pRGEB32–2) displayed an 1 kb size difference relative to pRGEB32–1 (Fig. 8a), consistent with the expected outcome. The correct band was gel-purified to obtain the linear backbone for subsequent ligation. Sanger sequencing of the resulting construct matched the designed sequence, confirming successful construction of a CRISPR/Cas9 genome-editing vector driven by the kenaf *HcU6–14P* promoter and targeting *HcALS*, which was designated HcU6–14P::HcALS-sgRNA-Cas9.

**Figure 9:**
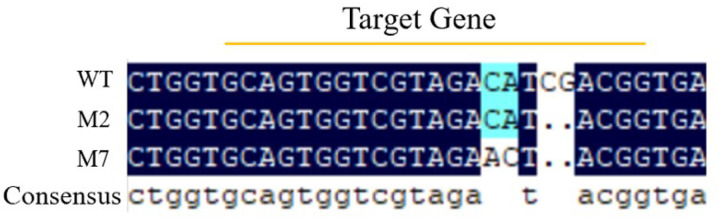
Mutation of HcALS was tested by sequencing. Sequencing results showed that two regenerated hairy root lines (named M2 and M7) had heterozygous mutations, with a mutation rate of 28.5%. The mutations included deletions and substitutions: in M2, the bases C and G were deleted; in M7, the bases C and G were deleted, and C and A were substituted with A and C.

Kenaf cotyledonary-node transformation mediated by *Agrobacterium rhizogenes* K599 generated seven regenerated transgenic hairy root lines. Using genomic DNA from regenerated hairy roots as the template, PCR amplification with the universal vector primer RTCas9 (Table 1) showed that all seven lines were positive (Fig. 8b), yielding a positive rate of 100.0%. Genomic DNA from PCR-positive hairy roots was further amplified with the target-site–spanning primers ALStest-F and ALStest-R, and the PCR products were subjected to Sanger sequencing. The sequencing results revealed heterozygous mutations in two regenerated hairy root lines, designated M2 and M7, corresponding to a mutation rate of 28.5%. The detected mutation types included deletions and substitutions. Specifically, line M2 exhibited deletion of the bases C and G, whereas line M7 showed deletion of the bases C and G, along with substitution of C and A to A and C (Fig. 9).

The CRISPR/Cas9 construct carrying the cotton *GhU6–9P* promoter and the *HcALS* target sequence was designated GhU6–9P::HcALS-sgRNA-Cas9. Using an *Agrobacterium rhizogenes* K599–mediated cotyledonary-node transformation system in kenaf, seven regenerated transgenic hairy root lines were obtained, among which five lines were confirmed to harbor the Cas9 cassette (Fig. 8b), corresponding to a positive rate of 71.4%. Compared with the kenaf HcU6–14P-based construct, the GhU6–9P-based vector exhibited a lower positive rate. Furthermore, amplification and sequencing of the target region using the site-spanning primers *ALS* test-F and *ALS* test-R detected no nucleotide mutations, indicating that, during genetic transformation of kenaf hairy roots, the transcriptional activity of the kenaf *U6* promoter is higher than that of the cotton *U6* promoter. Collectively, these results demonstrate that the kenaf hairy root genetic transformation system established in this study is robust and reliable.

## 4. Discussion

In this study, four endogenous U6 promoters derived from kenaf genome were successfully cloned. These promoters contain two typical conserved sequences, TATA box and USE element, and the distance between them is relatively stable. This structural feature is highly consistent with the endogenous U6 promoters reported in crops such as cotton, tomato, apple and eggplant[21]. In the CRISPR / Cas9 gene editing system, due to the long coding sequence of Cas9 protein[22], the reasonable simplification of other functional elements in the vector construction process helps to reduce the number of restrictive enzyme cutting sites, reduce the construction complexity, and to a certain extent, improve the practicability and efficiency of the gene editing system. Transfer PCR has been used to truncate the GbU6 promoter of Gossypium barbadense to varying degrees. The results showed that the effective transcriptional activity could be maintained even if the promoter length was shortened to 105 bp[23]. Similar conclusions have also been verified in plants such as sweet potato, tomato, apple and corn[3, 24–26], indicating that the U6 promoter has good tolerance to length changes in different plant species.

The above results show that the U6 promoter is truncated within a certain range, and moderately shortening the promoter length may have a positive effect on its transcriptional activity. In this study, *HcU6–14P* successfully drove the expression of sgRNA and achieved site-directed editing of *ALS* target genes in the kenaf genome. It is worth noting that the length of the *HcU6–14P* promoter fragment is 953 bp. Compared with the reported functional U6 promoter, it is still in a long interval. Whether it has the possibility of further truncation and maintaining or enhancing transcriptional activity remains to be verified by subsequent studies. In addition, the results of this study suggest that the endogenous U6 promoter of kenaf has certain tunability under different length conditions, which provides a potential strategy for the optimization of promoter elements in CRISPR / Cas9 vector. In the process of gene editing system construction, reasonable selection of promoter length may help to further improve the transcription level of sgRNA, thereby enhancing editing efficiency.

Further comparison showed that the gene editing system based on the endogenous HcU6–14P of kenaf obtained a mutation rate of 28.5% in hairy root transformation, while no target site mutation was detected in the positive hairy roots using the GhU6–9P promoter of cotton, indicating that the endogenous U6 promoter of kenaf showed higher functional adaptability in the genetic transformation system of kenaf. Previous studies have shown that the use of U6 promoters with consistent species origin can significantly increase the expression level of sgRNA, thereby improving gene editing efficiency[27, 28]. At present, the CRISPR / Cas9 system based on the natural U6 promoter has been successfully established in many plants such as Arabidopsis thaliana, cotton, rice and banana[29, 30]. Taken together, the results of this study further supported the important role of species-specific endogenous U6 promoter in the construction of CRISPR / Cas9 vector and the regulation of gene editing efficiency, and provided experimental basis for the optimization of gene editing system in kenaf and other crops.

## 5. Conclusion

In this study, the transcriptional activity of endogenous U6 promoter in kenaf genome was cloned and verified. The results showed that the endogenous U6 promoter *HcU6–14P* of kenaf could effectively drive the expression of sgRNA, and the site-directed editing of *ALS* target gene was successfully achieved in the hairy root transformation system of kenaf, with a mutation rate of 28.5%. In contrast, no target site mutation was detected in the sgRNA-expressing positive hairy roots driven by the cotton U6 promoter *GhU6–9P*. The above results indicate that the endogenous U6 promoter of kenaf has higher functional effectiveness in the CRISPR / Cas9 gene editing system of kenaf.

## Figures and Tables

**Table 1:** Primers used in this study

Primer name	Forward sequence(5’−3’)	Reverse sequence(5’−3’)
HcU6-1P	ATCTGTCGTCCGAGCTTAGC	CATAAGTAAACAGAGGCAGCG
HcU6-2P	GCTCTCTCCAACGTAGACTGC	AAACAGAGATAGAGATGGCAAA
HcU6-14P	TCCGGGCTTAGCAATATGAGC	ATGGTCTGAAGCTAACTCGATCTC
HcU6-15P	TGATCTAGGGATGGACCTTGGC	AAACACGATGGAGATGAGCACC
AtU6-26P	AAGCTTTCGTTGAACAACGG	TCAAAAATTATATCCTGTGGTCG
GbU6-9P	TTAATCTGATGCTCCACCTGC	ATGGCAACTTATCAAAGTTATTT
HcU6-1P-a	ATCTGTCGTCCGAGCTTAGCCGCCAAGCTT	CATAAGTAAACAGAGGCAGCGGTCTCCTGCC
HcU6-14P-a	TCCGGGCTTAGCAATATGAGCCGCCAAGCTT	ATGGTCTGAAGCTAACTCGATCTCGTCTCCTGCC
HcU6-14P-b	ATGATTACGCCAAGCTTATTCTCCGGGCTTAGCAATATG	AACTTCAGCTCTAAAAC**TCGATGTCTACGACCACTG**CTCTGAAGCTAACTCGATC
GbU6-9P-b	ATGATTACGCCAAGCTTATTCTTAATCTGATGCTCCACC	AACTTCAGCTCTAAAAC**TCGATGTCTACGACCACTGC**GCAACTTATCAAAGTTAT
RTCas9	GAGAATGAAGCGGATCGAA	CACACTCTTCAGTTTCTTGG
ALStest	ACGGCTTTAGGCTTCTTGGG	TTTGTTGGTCGCCGTTAGGA

Note: The nucleotide sequences of receptor plasmid in the primers were underlined. The reverse complementary sequences of *HcALS* target site were in bold.

**Table 2:** Seamless cloning reaction system

Component	Dosage(μL)
linearized vector	XμL
insert fragment	YμL
5×CE II Buffer	4μL
Exnase II	2μL
ddH_2_O	to 20

Note: X = [0.02 × number of base pairs of the cloning vector] ng (0.03 pmol); Y = [0.04 × number of base pairs of the insert fragment] ng (0.06 pmol)

**Table 3:** LR reaction system

Component	Dosage(μL)
PRNTR-U6	2μL
PGWB533	2μL
LR recombinase	1μL
Total	5μL

## Data Availability

The datasets analyzed in this study are available upon request.
